# The dynamics of seated shot put: a case study example of pole grip optimisation

**DOI:** 10.3389/fspor.2025.1509435

**Published:** 2025-01-20

**Authors:** Connor J. Holdback, Rony Ibrahim, David S. Haydon, Paul Grimshaw, Richard Kelso, Ross A. Pinder

**Affiliations:** ^1^ School of Electrical and Mechanical Engineering, University of Adelaide, SA, Australia; ^2^ College of Sport Sciences, Qatar University, Doha, Qatar; ^3^ South Australian Sports Institute, Adelaide, SA, Australia; ^4^ Athletics Australia, Adelaide, SA, Australia; ^5^ College of Health and Life Sciences, Hamad Bin Khalifa University, Doha, Qatar; ^6^ Allied Health & Human Performance, University of South Australia, Adelaide, SA, Australia; ^7^ Performance Insights & Innovation, Paralympics Australia, Adelaide, SA, Australia

**Keywords:** paralympic, sport, disability, biomechanics, seated throw

## Abstract

The impact of pole position in seated shot put has been a key research question both in the previous literature and for coaches in the field. The aims of this research were to understand the dynamics of seated shot put and to investigate the impact of changing pole grip height on trunk parameters. Three grip heights are compared: the athlete's standard grip, 75 mm higher than standard, and 75 mm lower than standard, to determine which grip produced greater angular velocity and power at the trunk. In addition, a post-analysis intervention was implemented following this investigation where the athlete completed four weeks of training (8 sessions) with a grip height that was indicated to be superior. The trunk was found to provide the largest contribution to the movement, with a 64% of the total velocity contribution. A lower grip height was found to generate greater power and velocity in trunk rotation and flexion when compared to higher grip heights. When assessed over the intervention period, the lower grip height showed an increased rate of improvement throughout, and a performance advantage over the standard grip after just four sessions. This research demonstrates that pole grip height can impact athlete biomechanical parameters and may improve overall performance given sufficient time.

## 1 Introduction

Seated shot put features a total of 11 medal events at the Paralympic Games, positioning it as a key discipline for the success of national teams. Many athletes utilise a throwing pole in seated shot put for support and power production. This presents a significant performance advantage for some classes, such as for the F32 class in Tokyo 2020, where these pole users performed significantly better than non-pole users in the same class (10.8 ± 0.5 m and 8.7 ± 0.2 m respectively) (1). At this same competition, a range of different throwing pole positions and grips were used by the athletes, where many medal-winning athletes were seen to use a pole differently, even within the same class. Some level of variability between classes may be primarily explained by differences in impairment, but the level of variability seen between medal winners within the same class is perhaps indicative of the throwing pole not being a well-understood equipment variable in seated shot put at the highest level.

To date there has been no published research that investigates the impact of the throwing pole on the dynamics of seated shot put, which is in part due to the complexity of measuring pole forces (2). O'Riordan et al. (3) investigated the kinematic effect of changing the position of the throwing pole using two positions: a “standard” position (typically used by the athlete) and a “closer” pole position (5 cm closer to the athlete). From this research, the closer pole position resulted in greater trunk rotational velocity but throw performance was not improved (standard = 8.84 ± 0.34 m, closer = 8.86 ± 0.36 m). However, the effect on trunk rotation is notable, as it has been shown in an earlier study on female shot putters that increased trunk velocities can lead to improved throwing performance (4). It could be speculated that the absence of performance improvement seen using the new pole position may be due to motor learning limitations in a short time period, where a potentially advantageous but novel position does not produce a better performance initially due to imperfect coordination (5). Thus, a change in position should be evaluated over an extended period to allow for training adaptations and realisation of the performance potential. Since changes in pole position have been seen to have an impact on throwing kinematics (3), it is important to understand the joint dynamics behind such results.

A change in pole position requires mechanical changes to the athlete's equipment, providing a barrier to the athlete's ability to experiment with new positions. A more commonly manipulated variable in practice is the pole grip height. However, no previous research has looked at its effect in seated shot put. Therefore, the aim of this research was to conduct a biomechanical analysis case study to understand the kinetics of seated shot put and investigate the impact of changing pole grip height on trunk parameters. It is proposed, based on the prior research, that a change in pole grip height is likely to influence the angular velocity and power of the trunk.

If it is seen that a change in grip height presents advantageous changes in the biomechanical dynamics of the athlete, the research aims to understand how this information can inform a pole grip change that could improve the performance of an athlete over time by conducting an intervention over an extended training period. It is hypothesised that a better pole grip height may require an adjustment period whereby the athlete can explore the capabilities of this positional change before producing improvements in performance.

## 2 Methods—biomechanical analysis

### 2.1 Study design and data collection

An elite female (F34) seated shot put athlete was recruited in collaboration with their National Sporting Organisation, where this athlete (and their coach) was identified as being interested in equipment optimisation. Informed consent was given by the athlete in accordance with the approved Human Ethics application (H-2022-147). The athlete presented with a high level of fitness, and they reported no injuries or recent changes to their equipment. The testing took place in a large, covered training area to control for weather conditions. For context regarding pole use in this athlete's classification, the majority (≈60%) of athletes in the F34 classification used a pole at the Tokyo 2020 Paralympic Games, where two out of the three medallists used a pole (1).

**Figure 1:**
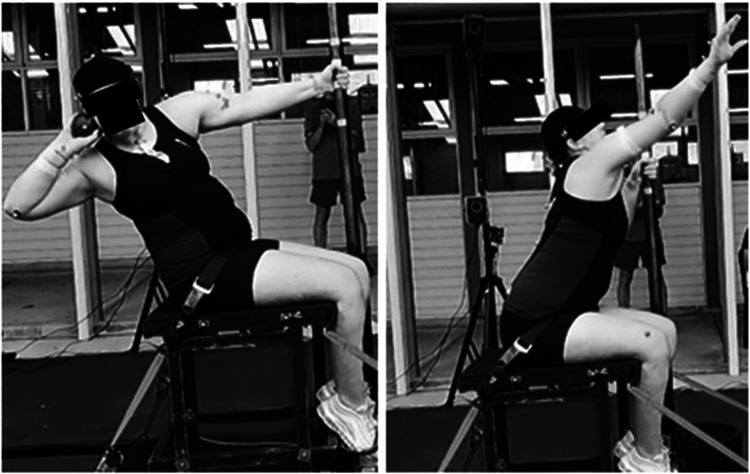
Images of the athlete performing a throw with the instrumented pole and reflective markers. These also provide a demonstration of the defined start and end of the throw; the Power Position (left), and Release (right). Presented with permission.

After performing their standard warm-up, formal testing began where the athlete performed three throws with three pole grip heights: the standard grip used by the athlete (seen in Figure 1), a higher grip (standard +75 mm), and a lower grip (standard −75 mm) for a total of 9 throws. Throws with these pole grips were performed in a random order so as not to bias any one position, and the athlete was asked to rest between throws such that they could produce their best possible performance in the next attempt. A further three throws with the standard grip were performed at the end to act as a control for quantifying fatigue. No coaching cues were allowed during the formal testing; the only instruction to the athlete was to attempt to throw as far as possible. The change of 75 mm was selected as this provided a substantial change to the hand position while remaining within a comfortable range for the athlete, based on feedback from experienced coaches and the athlete. The athlete used their own throwing frame (seat) with their pole removed and an instrumented pole placed in the same relative position. This allowed the athlete to use their normal strapping and foot supports (as seen in Figure 1). The instrumented pole that was used during the testing incorporated a load cell (ME Systeme K3D120 1000 Hz) for measuring pole forces during the throw as outlined in previous research (2). The standard shot implement was used for this athlete (3 kg) as would be used for their class in competition.

A Vicon motion capture system with twelve V8 Vantage cameras was used to capture at 200 Hz the upper body kinematics of the athlete. Anatomical landmark markers (23 in total) were used to track the upper limbs, head, and trunk (Table A1). Accurate tracking of the pelvis during the trials was challenging due to the athlete's interaction with the seat and supportive strapping. To accommodate this, four additional markers were placed on the throwing frame as a reference for the seat. The pelvis was modelled as a segment restrained to the seat with 3 degrees of translation informed by a single marker at the lumbosacral joint (L5S1).

### 2.2 Kinematics

Segment coordinate systems were established from corresponding markers (6). The athlete was then modelled as a “linked segment model” (7), with segment inertia calculated using the methods outlined by De Leva (8). Additional details regarding model modifications can be found elsewhere (9, 10). Joint rotations were defined in the local coordinate system of the proximal segment, and trunk rotation about the pelvis was defined with L5S1 as the joint centre.

The athlete was modelled as a chain of linked rigid segments with L5S1 as the proximal point and the throwing hand as the distal endpoint. Linear velocity of the endpoint ($V_{s}$) is considered as a sum of the linear velocity of the proximal point ($V_{L5S1}$), the cross product of the trunk segment angular velocity ($\omega_{T}$) with its relative moment arm ($r_{L5S1,S}$), and the cross product of the shoulder, elbow and wrist angular velocities ($\omega_{j}$) with their corresponding moment arms ($r_{\;jc,S}$):

(1) $$V_{S}=V_{L5S1}+\omega_{T}\times r_{L5S1,S}+\overset{q}{\underset{\;j=1}{\sum }}\;\omega_{j}\times r_{\;jc,S}$$

where $r_{\;jc,S}$ is the position vector from the respective joint centre to the distal endpoint. In addition to calculating the kinematic contribution of each joint to the resultant shot velocity, this approach allows end-to-end validation of the linked segment model by comparing the left and right sides of the equation above, i.e., by comparing the calculated end-effector velocity from joint contributions to the measured end-effector velocity (11). This is then presented as the joint contributions to the endpoint linear velocity in the direction of the throw (horizontally), to better indicate contributions to throw distance. Due to the complex interaction between the hand and the shot implement during release, the endpoint is defined to be the midpoint between the second and fifth knuckle of the throwing hand.

The definition of joint velocity contribution allows insight into which joint may have the largest impact on throw velocity and thus throw performance. Such results can provide guidance as to what aspect of the athlete's biomechanics should be the focus of detailed investigation. This is particularly helpful for movements involving many joints where the relationships between a position change (grip height) and technique may be complex.

### 2.3 Kinetics

Forces exerted on the pole by the athlete during the throw were measured using an instrumented pole incorporating a load cell as per Holdback et al. (2). Together, the measured pole forces and upper-body kinematics (i.e., wrist, elbow, shoulder, and trunk L5S1) enabled the calculation of joint moments through inverse dynamics equations outlined by Kingma et al. (9). Detailed descriptions of the 3-D inverse dynamics model used are available in Faber et al. (12, 13), Ibrahim et al. (11), and Kingma et al. (9).

### 2.4 Data analysis

The resulting mean angular velocity, torque, and power of each joint were presented with the calculated standard error (SE) to visualise trends (14). The beginning of the throw was defined as the power position, indicated by a peak in the measured pole forces which coincided with the athlete's trunk segment velocity being zero in the posture depicted in Figure 1. The end of the throw was defined as the point of release, indicated by the peak wrist joint angular velocity. The start and end points were chosen as objective indications present in the data to maintain consistency amongst all trials.

## 3 Results—biomechanical analysis

### 3.1 Joint angular velocity and torque

**Figure 2:**
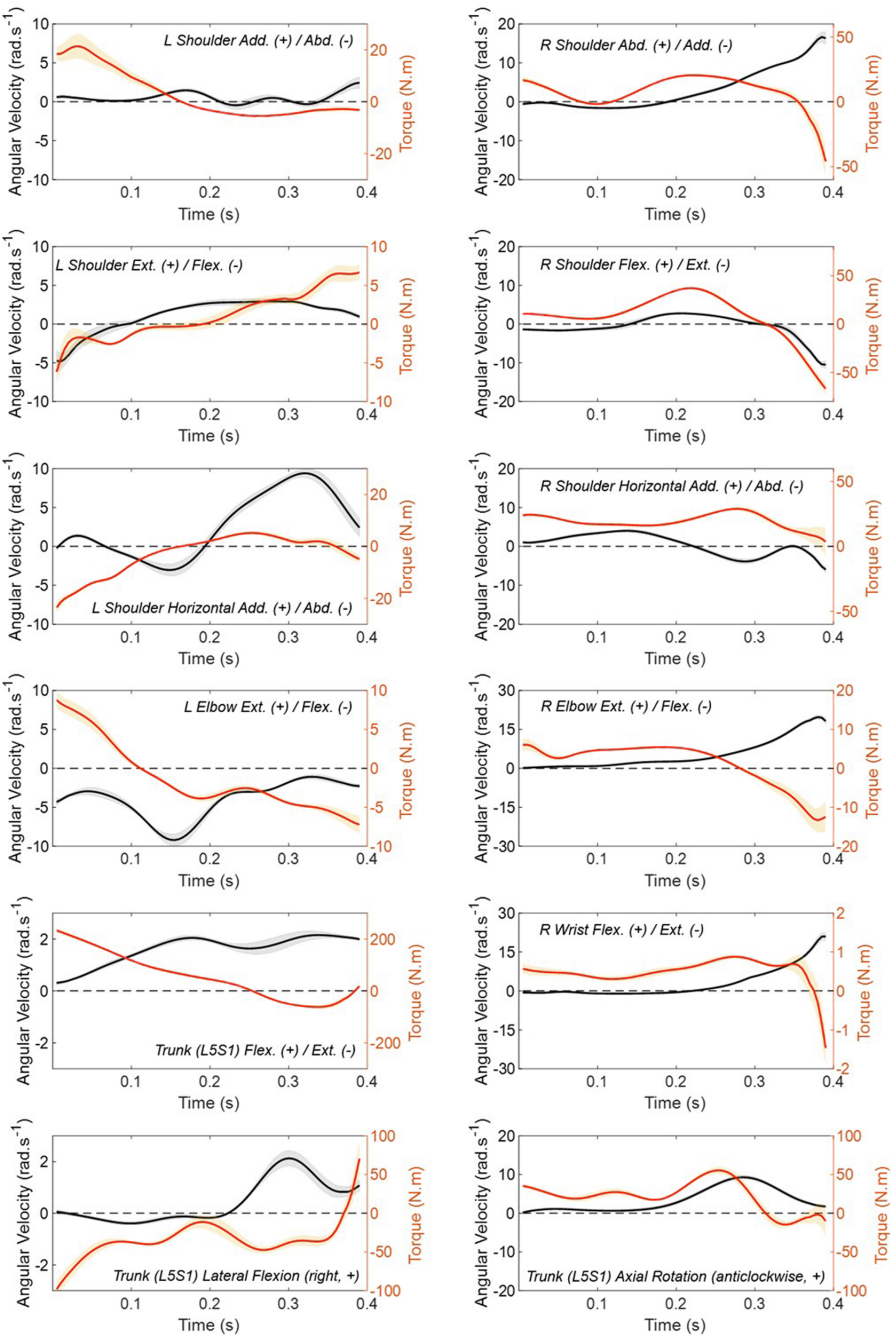
Joint angular velocity (rad s^−1^) and torque (N m) for seated shotput performed with the standard grip. Right arm is the throwing arm, left arm is holding the pole. Measured from the power position (t=0) to release (t≈0.4). Here, the truncation “Abd.”, abduction, and “Add.”, adduction.

Joint parameters were investigated with the athlete's standard grip to first understand the biomechanics behind their throw. Time series data for mean angular velocity and torque for each joint are presented in Figure 2 with standard error (SE) shading. Distal segments are seen to achieve incrementally higher velocities than proximal segments in the throwing arm (R), but not necessarily in the pole arm (L). Proximal segments are seen to produce greater joint torques than distal segments, in alignment with expectation. Notably, the highest torque observed is for trunk flexion (238 ± 15 N·m) at the beginning of the movement.

To summarise emergent movement patterns, the throwing arm (R) exhibited activation in shoulder flexion (39 ± 1.1 N·m) and abduction (21 ± 1.4 N·m) in the mid phase of the movement together with elbow extension (5.5 ± 0.32 N·m), in addition to a steady contribution of shoulder horizontal adduction (18 ± 2.5 N·m) throughout. Interestingly, high velocity activation in shoulder extension (65 ± 6.6 N·m) is seen in the late phase of the movement, resembling a downwards “flick” just prior to release. It is possible that this “flick” movement was more pronounced than expected due to the relatively light shot mass (3 kg) used in the F34 seated shot put class. The pole arm (L) produced an initial torque predominantly via shoulder adduction (22 ± 6.5 N·m) followed by elbow flexion (8.3 ± 1.0 N·m) and an active high velocity contribution from shoulder horizontal adduction (4.8 ± 0.35 N·m) in the late phase of the movement, likely supporting trunk rotation prior to release.

### 3.2 Joint contributions

**Figure 3:**
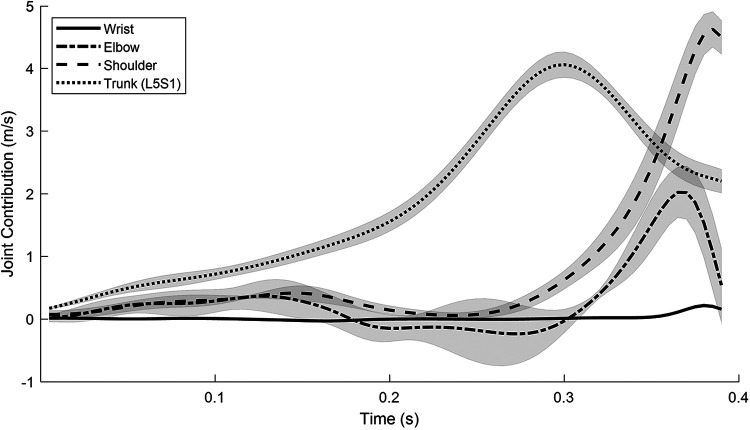
Joint angular velocity contribution to horizontal shot velocity—from the power position (t=0) to release (t≈0.4), as recorded for trials with the standard pole grip.

The kinematic contribution of each joint to the horizontal shot velocity is shown in Figure 3 to highlight the relative joint contributions at different points throughout the throw. The calculated velocity contribution can be compared to the measured end-effector velocity to determine the error present in the link segment model (Equation 1). Here the root mean squared error (RMSE) is 3.7%, indicating adequate congruency in the model. The trunk is the largest contributor with a mean relative contribution of 64% over the duration of the throw. The wrist mean contribution to the shot velocity is seen to be relatively low, likely due to the small segment length relative to the other segments.

### 3.3 Trunk kinematics and kinetics

**Figure 4:**
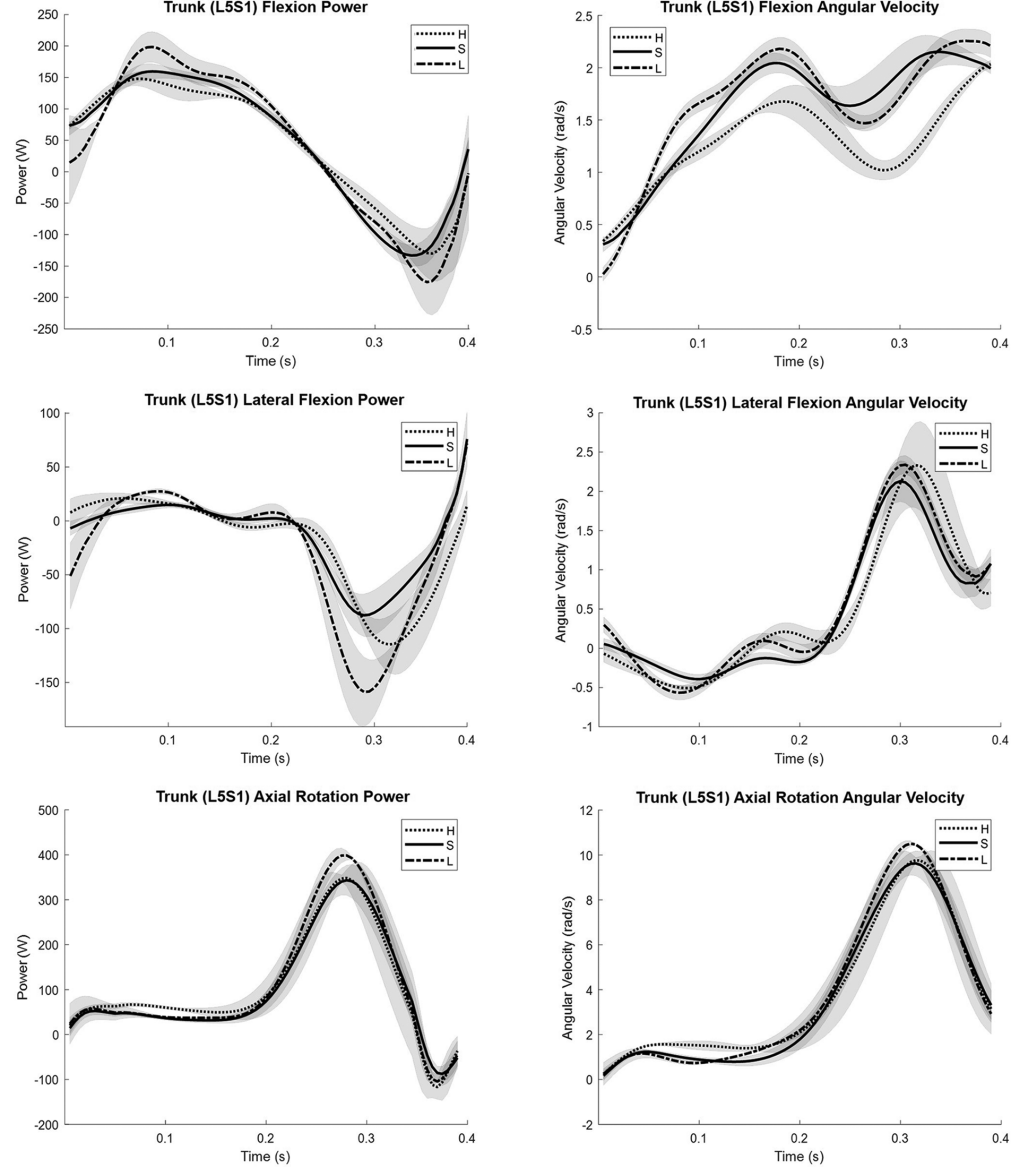
Trunk (L5S1) joint power and angular velocity for the standard pole grip height (S), low grip (L), and high grip (H) measured from the power position (t=0) to release (t≈0.4).

Due to the large relative contribution of the trunk, along with the implications from prior research showing that pole position can impact trunk angular velocity (3), a closer inspection of trunk parameters is warranted. To best understand each grip's potential for performance, trunk power is presented for different pole grip heights in Figure 4. Here, power production in both flexion and rotation were substantial for all grip heights, where flexion power was seen to peak in the early phase of the movement, and axial rotation power in the latter phase of the movement. Peak angular velocity in trunk axial rotation is found to be much higher than trunk flexion, which is more consistent throughout. Very little power production (positive power) was seen in lateral flexion, and together with a relatively low angular velocity, this could imply that lateral flexion acts more in a stabilisation role rather than productive work. When comparing different pole grip heights, the lower grip exhibits the greatest trunk axial rotation power and associated angular velocity when compared to the other grip heights, and this is similarly the case with trunk flexion.

## 4 Methods—post analysis intervention

### 4.1 Intervention definition

These findings indicating greater trunk angular velocity and power, together with the prior literature presented by Judge et al. (4) suggest that improvements in trunk parameters can lead to improved performance. Subsequently, this led to the conclusion that a lower grip should be tested over an extended period of time in order to evaluate its potential to improve performance in this athlete. This, when presented to the athlete and coaching staff, resulted in a recognition that the overall increase in trunk performance as seen with the lower grip height could be advantageous for potential gains in throwing performance. Following further discussion and collaboration, the lower grip was tested over a four-week training period consisting of 8 sessions in total. The duration of four weeks was dictated by the athlete's competition schedule.

### 4.2 Intervention design

To evaluate this new position, the athlete undertook the following four-week training period comparing their standard grip and the new lower pole grip height. In this testing period, the athlete performed three throws with both their standard grip and the lower pole grip height at the beginning of every training session following their normal warm up, and the distances thrown in each position were recorded. The standard grip was kept in the program to control for athlete readiness and conditioning over the training period. The grip height performed first was alternated between sessions so that neither grip height would gain an advantage due to a change in the athlete's level of readiness to perform between the first and last three throws. In this case, grip heights were not alternated between every throw to provide the athlete more familiarity such that they can produce the best throw possible with that grip.

### 4.3 Statistical analysis

To provide an objective measure of performance, a trend line of the best throw performances in each grip position per session was assessed. The choice to display the trend line for the best throw performances in each position rather than the mean was decided upon, since this is the measure of performance that ultimately determines success in competition, and thus best demonstrates the potential of each position in the long term. This approach also rejects the larger variation initially expected from a novel position. Similarly, a logarithmic trend line was selected as it best represents the expected asymptotic trajectory of performance where improvement is faster initially (15, 16).

## 5 Results—post analysis intervention

### 5.1 Intervention findings

**Figure 5:**
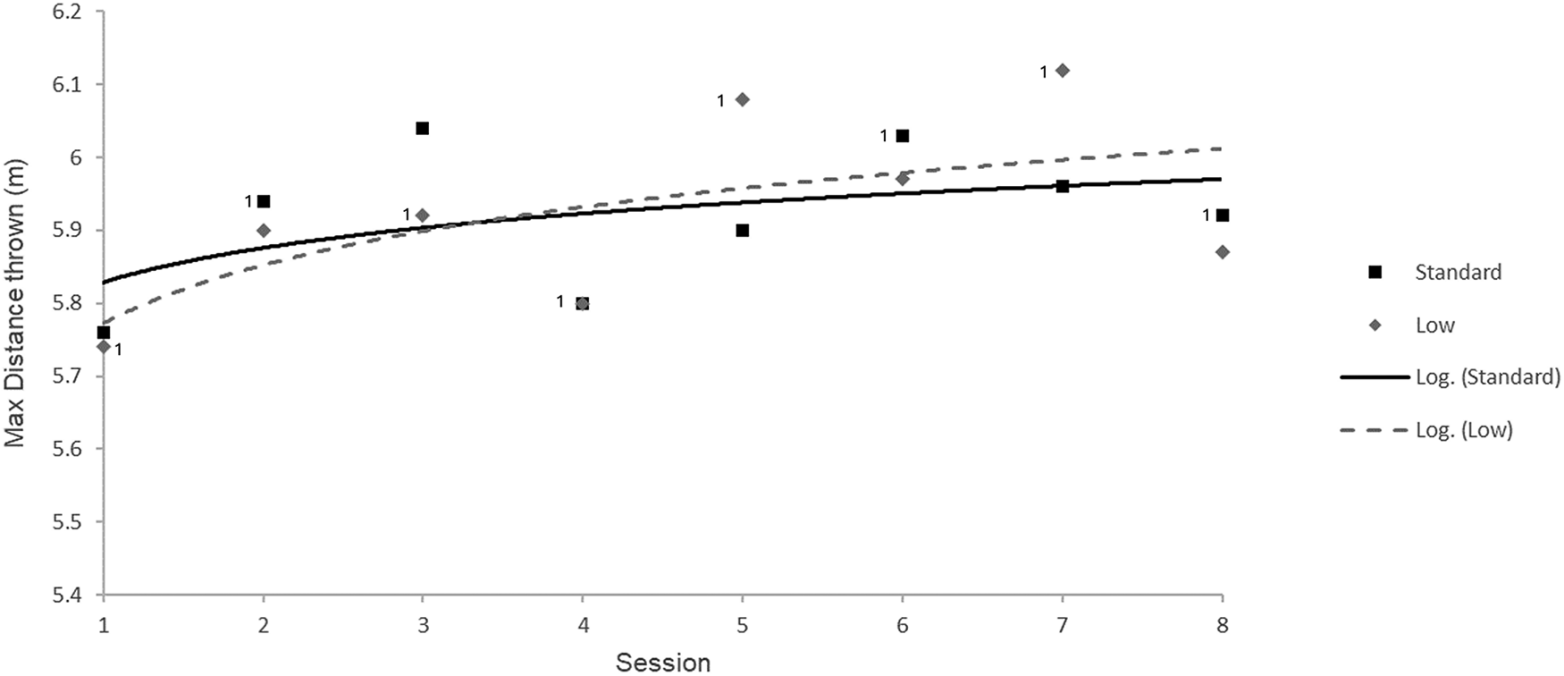
Best throwing performance for each grip during the intervention period with logarithmic trend lines (R_Low_ = 0.62, R_Std_ = 0.48). ^1^ Indicates which position was performed first in that session.

The best throwing performances for each session and grip height during the intervention period are shown in Figure 5. The standard position consistently outperformed the lower grip height position in the first three sessions; after this, the grip height used first in the session largely dictated the best performance in that session. However, in the latter half of the training period, the lower grip position outperformed the standard grip with a greater margin when performed first. The potential seen in the new lower grip position is further demonstrated by the two best performances of the entire testing period thrown using this new position (session 5 and 7). The standard deviation observed was larger for the low grip height as expected (15.5 cm vs. 8.3 cm for the standard grip). There was no distinguishable point where the low grip performance appeared to plateau within the testing time, and the standard deviation did not reduce to a similar level to that of the standard position, which may also indicate that the adjustment period was ongoing. From this, it seems the potential of the new position was not realised within the timeframe of this study. Ultimately, the resultant trend line shows a faster improvement for the new low position and an advantage over the standard grip after four sessions.

## 6 Discussion

### 6.1 Trunk kinematics and kinetics

This research aimed to provide insight into the kinematics and kinetics of seated shot put and investigate the impact of changing pole grip height on trunk parameters in a single athlete (case study). Regarding the kinematics and kinetics, distal segments were seen to reach greater angular velocities, while proximal segments were seen to produce greater joint torques, where the highest torques were observed in the trunk. When assessing the contribution of each joint to the shot velocity, the trunk also produced the largest mean contribution, followed by the shoulder (21%), elbow, then wrist. When comparing different pole grip heights, the lower grip led to greater power and angular velocity in both trunk flexion and axial rotation, which suggests the original proposition, that a change in pole grip would impact trunk parameters, can be accepted. The observed increase in trunk rotational velocity is in alignment with the results for the closer pole position in the study by O'Riordan et al. (3). In that study, it was speculated that the increase may be due to the greater range of motion (ROM) that can be achieved in this position. It is possible that the lower grip tested in the present study is producing a similar effect as it may also enable an increased ROM. In fact, this is what was seen: the lower grip had more trunk flexion-extension ROM on average than both the standard and higher grip (+4° and +9° respectively). This adds credibility to the notion that the impact of pole position on ROM may be a factor to consider when determining optimal pole placement for athletes. Although it was not the aim of this testing to compare the outright performances between different pole grips, it may be valuable to report that there were no significant differences seen in the performance between the different pole grips (Low = 6.15 ± 0.27 m, Std = 6.18 ± 0.25 m, Hi = 6.18 ± 0.10 m).

### 6.2 Intervention results

In addition to the first aim of this study, which was to provide insight into the kinematics and kinetics of seated shot put, a second aim was developed to understand how this can inform a pole grip change that could improve the performance of an athlete over time. This second aim was achieved by conducting an intervention with a new pole grip position over an extended training period. The new lower pole grip initially performed worse than the standard pole grip in the first 4 sessions, after which the lower pole grip began to perform better as indicated by the trend line in Figure 5. This suggests the second proposition, that performance with a better pole grip height may overshoot that of a previous grip height after adjustment period, can be accepted. The confidence in the trend line is limited by the modest R-values of 0.62 and 0.48 for the low and standard grip, respectively. This is in part due to the sporadic changes in performance between sessions as a result of uncontrollable external factors (e.g., environmental conditions, recovery, etc.), but also due to the performance impact observed based on whether the position was performed first or second in the session. However, other observations improve the confidence that the lower pole grip presents potential over the standard grip, such as the two best performances across the eight sessions having used the lower grip.

### 6.3 Practical implications

This is the first time the kinetics of seated shot put have been presented in the biomechanical research, thus providing an opportunity to suggest some implications of this early research for practitioners. For example, active torque contribution from shoulder adduction and horizontal adduction in the pole arm, as well as elbow flexion, indicate that increasing strength in those areas may support throw performance. As for the throwing arm, in addition to seeking strength in shoulder flexion and horizontal adduction, as is typical in throwing, also including exercises to support shoulder extension may enhance the flick motion observed in the late phase of the throw. Perhaps most importantly, the magnitudes of trunk torque and power observed in this athlete may imply a large relative importance of improving strength in this area, specifically in trunk rotation and flexion. However, there may be a lateral stability component that remains important as indicated by the notable torque observed opposing lateral flexion. This suggests that placing a focus on flexion and rotation of the trunk in the athlete's strength and conditioning program may be valuable for improving throw performance. This suggestion is strengthened by the promising intervention results where the position that maximised trunk power was indicated to provide greater potential for improved performance. It is critical to consider that these results are based on a single athlete, however as discussed above, these results are in agreement with the other case study performed in seated shot put with different pole positions by O'Riordan et al. (3). It should be noted that resultant performance is dependent on the entire kinetic chain (17), and this should be considered when attempting to focus on a single component in training, especially since previous literature has shown the important relationship between all components of the kinetic chain in rapid throwing movements (18–21). From the results above it is clear the shoulder also has a large contribution to the movement (21%), suggesting it is a meaningful contributor alongside the trunk.

Further insight regarding the adaptation period is gained from the intervention period where, for this athlete, four weeks was sufficient to see improved performance from a novel position but was likely insufficient to realise its full potential. This may provide guidance for coaches and athletes on how long to trial new positions and how soon to begin a trial prior to major competition.

The following practical implications may be taken from this study, with the knowledge that this is a single athlete and thus caution should be taken regarding generalisability to other athletes:

• The trunk provides a large contribution relative to other segments in seated shot put, making it a candidate for special attention in an athlete's strength and conditioning program.
• Pole grip may impact trunk range of motion, angular velocity and power. Seeking a pole grip that maximises such parameters is indicated to predict a better pole position for that athlete.
• In the absence of precise motion capture equipment and a force sensing pole, maximising the trunk range of motion could be used as an informative proxy for seeking a better pole position.
• Four weeks (8 sessions) showed some improvement in performance when using a new pole position, but it was not long enough to fully realise the position's potential for this athlete. This may be informative when planning a pole grip change in other athletes.

### 6.4 Limitations

Biomechanical modelling is complex, where assumptions are often made to simplify the human body into rigid segments linked by simple joints. In this study, the resultant linked-segment model was tested for congruency by comparing the calculated end-effector velocity from joint contributions to the measured end-effector velocity. Here a RMSE of 3.7% provided adequate confidence in the model. Body segment inertias were calculated using De Leva's adjustments to Zatsiorsky-Seluyanov's model (1996), providing a generally accepted input for calculating segment parameters for linked-segment modelling. The resultant joint power from inverse dynamics analysis provided insight into the measured joint angular velocities that appeared logical and informative. Based on this, it is expected that the modelling performed in this study produced accurate results.

Study designs involving acute testing of novel positions in a single session may present a limitation where the athlete is unlikely to show performance improvement for anything other than their standard position in which they have the most familiarity. To investigate a new position for its effect on performance, a longer trial period is required. This is why a four-week training period was designed to compare the promising lower grip to the standard grip. This intervention included an in-subject control feature, whereby the athlete acted as their own control by performing three maximum effort throws in the new position as well as the standard position in each session. A potential issue was identified whereby the athlete may perform better in the first 3 throws when compared to the last 3, due to general preparedness. To address this, the grip height performed first was alternated between sessions. This approach was confirmed to be necessary in Figure 5, where it appeared that the position performed first was more likely to produce a better performance in that session. While this four-week testing period provided insight into the potential of a new position, it is limited by its short duration. This was due to the availability of the athlete close to their competition season. Therefore, a longer testing period is necessary to realise the full potential of a new position, ideally indicated by a reduction in throw performance standard deviation to a similar level to that seen with their standard position.

## 7 Conclusion

This research demonstrated that pole grip position can impact an athlete's biomechanical parameters and may improve overall performance given enough time. Specifically, changes in pole grip height were seen to impact the measured trunk angular velocity/power and selecting a pole position that maximises these trunk parameters was shown to predict a pole position that demonstrated improved performance for that athlete over a four-week period. However, it is important to note that these findings are based on a single athlete within a specific classification. As such, future work is required to investigate other athletes or a group of athletes to improve the generalisability of these findings. Group analysis will be challenging as is often the case in paralympic sport because impairment heterogeneity is so high. Regardless, this work provides insight into the throwing dynamics of an elite seated shot put athlete, as well as a framework for the optimisation of pole position for an individual athlete, where an individualised approach is likely the most effective avenue for paralympic athletes.

## Data Availability

The raw data supporting the conclusions of this article will be made available by the authors, without undue reservation.

## Appendix A

**Table A1:** Defining body segments and their anatomical landmarks used in the present study.

Body segments	Corresponding anatomical landmarks
Pelvis	Right and left antero-superior iliac spines; midpoint between the postero-superior iliac spines; navel
Abdomen	Navel; 12th thoracic (T12); xiphoid process
Thorax	Xiphoid process; 6th thoracic (T6); suprasternal; 7th cervical (C7);
Head	7th cervical (C7); right and left tragion; head vertex
Upper arm	Acromion process; lateral and medial humeral epicondyles
Forearm	Lateral and medial humeral epicondyles; radial and ulnar styloids
